# Recurrent Brain Abscess in a Child With Cyanotic Congenital Heart Disease

**DOI:** 10.7759/cureus.32618

**Published:** 2022-12-17

**Authors:** Adithya Kiran, Amar Taksande, Richa Chaudhary

**Affiliations:** 1 Department of Paediatrics, Jawaharlal Nehru Medical College, Datta Meghe Institute of Medical Sciences, Wardha, IND; 2 Department of Pediatrics, Jawaharlal Nehru Medical College, Datta Meghe Institute of Medical Sciences, Wardha, IND

**Keywords:** antimicrobial therapy, transposition of great arteries, recurrent, cyanotic heart disease, brain abscess

## Abstract

Brain abscesses are rare but life-threatening conditions associated with morbidity and mortality. They are encountered in unoperated and partially treated cyanotic congenital heart diseases. Our patient is a diagnosed case of transposition of great arteries with a ventricular septal defect. She had recurrent abscesses for which a combination of antimicrobial therapy and surgical excision was performed. Surgical excision carries a great risk of rupture of abscess into the ventricular system and is associated with poorer outcomes. The outcome of brain abscess primarily depends on Glasgow Coma Score (GCS) at the time of admission and ventricular extension of the abscess. Fortunately, our patient showed good results without any apparent neurological sequelae. Early diagnosis of the brain abscess and timely administration of antibiotics help in a good outcome.

## Introduction

Brain abscess is one of the most serious diseases of the central nervous system (CNS) [1]. They are rare but usually life-threatening conditions particularly encountered in developing countries. Brain abscess is a focal, intracerebral infection that begins as a localized area of cerebritis and develops into a collection of pus surrounded by a well-vascularized wall or capsule [1-4]. They can occur in children of any age but are mostly seen in children between the ages of four and eight years and in neonates [5]. Causes of brain abscesses include embolization due to cyanotic congenital heart disease and meningitis, and over half of the brain abscesses may result from sinusitis, otitis, or dental infections [1]. Head trauma due to penetrating injuries can implant a foreign body and can lead to brain abscess formation [6]. Immunosuppressed children (HIV/AIDS and childhood malignancies on chemotherapy) are at increased risk of developing brain abscesses. The mainstay of management includes a multidisciplinary approach involving a pediatrician, neurosurgeon, neuroradiologist and infectious disease specialist [7]. Herein, we report a seven-year-old female child who was diagnosed with transposition of great arteries (TGA) with large ventricular septal defect (VSD) with small atrial septal defect (ASD) with severe pulmonic stenosis (PS). She had recurrent brain abscesses in the past for which she was operated on twice two years ago. She now presented to the casualty with fever and right-sided focal seizures. CT and MRI scans were done and were suggestive of recurrent brain abscess. Craniotomy and excision of the abscess were done and the patient had a good recovery.

## Case presentation

**Figure 1:**
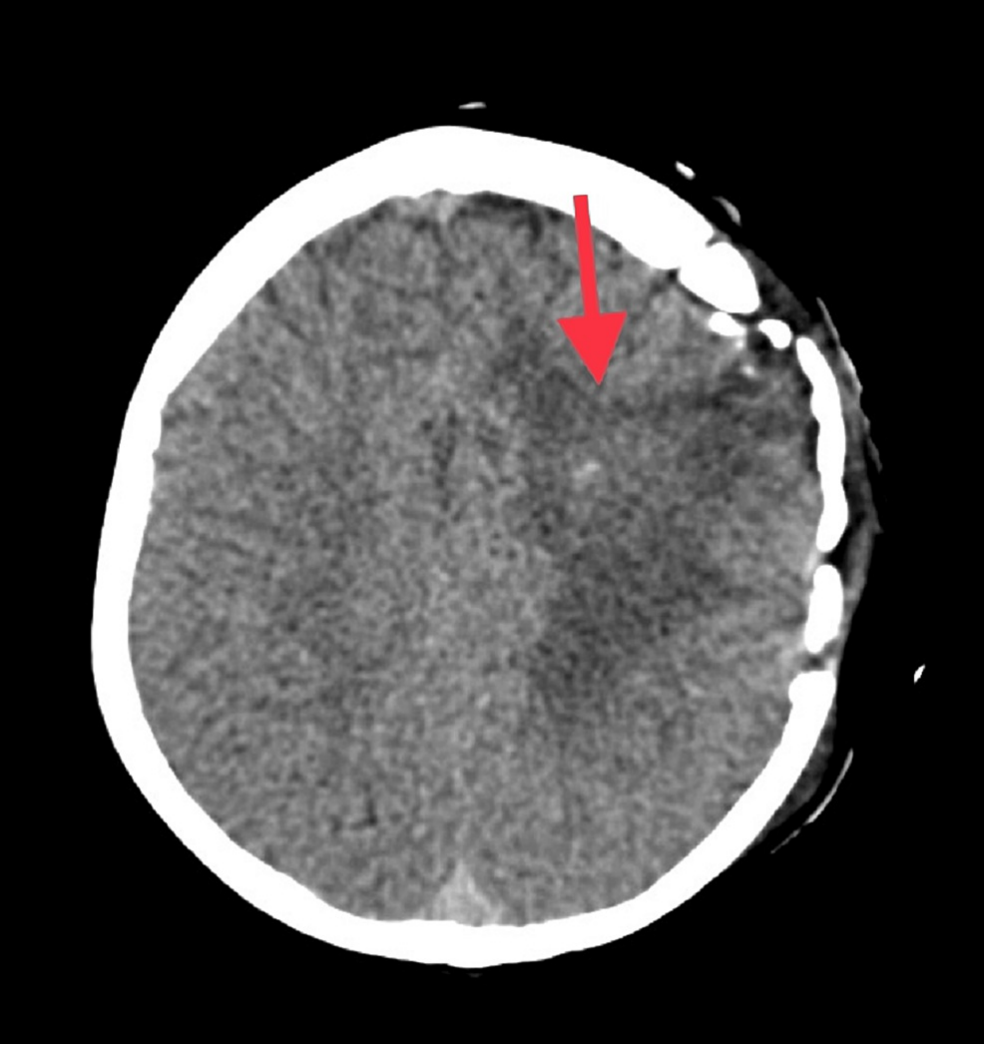
Axial non-contrast CT section showing post-operative oedema in the left frontoparietal lobe and adjacent post-operative calvarial defect

**Figure 2:**
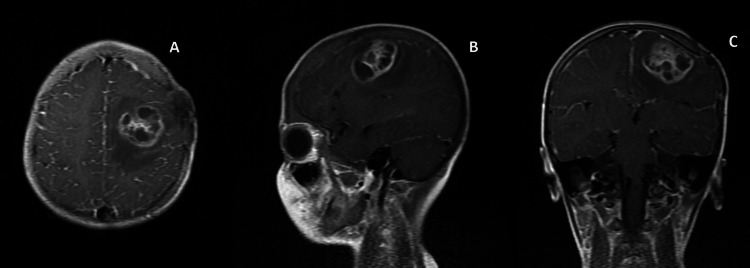
Axial (A), Saggital (B), Coronal (C) Contrast MRI brain showing a large, thick-walled, multi-septate peripheral enhancing lesion in the left high frontoparietal region measuring around 2.1 x 3.2 x 3.5 mm (CCxTRxAP) with associated perilesional oedema.

A seven-year-old girl with TGA with large VSD and small ASD diagnosed at two months of age, presented in the emergency department with fever and right-sided focal seizures. The complaints started three hours before the presentation when she had a high-grade fever for one day followed by numbness over the right arm and later eyeballs deviation towards the right side and altered consciousness. After 12 hours, the patient had focal convulsions involving the right side of the body. There was no history of vomiting, ear discharge, dental caries or otitis media. Once the patient arrived, intravenous (IV) midazolam (0.1 mg/kg) was given and the convulsions were aborted after 10 min. The patient was on home medications of levetiracetam and valproate for two years, with good compliance. She had her first seizure at five years of age when she was diagnosed to have a brain abscess in the left frontoparietal lobe measuring 3.5 mm for which craniotomy and aspiration of the abscess were done. She was treated with a course of intravenous broad-spectrum antibiotics for four weeks and then had another seizure associated with a fever. A repeat CT scan was done, which revealed postoperative haemorrhagic collection in the left frontoparietal lobes exiting to the left caudate nucleus along with surrounding white matter oedema (Figures 1, 2). She was treated with anticonvulsants levetiracetam and valproate and was discharged. The patient had no further convulsion episodes since then. Family history did not reveal members with a similar history. No history of consanguinity between her parents.

At the time of presentation, the patient was febrile (101ºF/38.3°C), blood pressure (96/60 mmHg), heart rate (132 beats/minute) and respiratory rate of (36/minute), which implies the patient had tachycardia and tachypnea. Her oxygen saturation (72% on room air) was similar to her baseline oxygen saturation range of 75%. Neurological examination showed generalised hyperreflexia, extensor plantar response, and decreased power on both the upper and lower limbs of the right side. Contrast-enhanced CT scan head showed residual abscess with surrounding white matter oedema and gliotic changes in the left frontal lobe involving the left caudate nucleus. In a blood examination, complete blood count showed Hb- 15.4, total leucocyte count of 15,000 per mm^3^, and C-reactive protein of 4.63 mg/ml were elevated slightly. A lumbar puncture was performed and cerebrospinal fluid (CSF) analysis revealed clear transparent fluid with a white cell count of 6/cumm and protein of 40 with glucose of 106. MRI of the brain with contrast showed a large thick-walled multiseptated peripheral enhancing lesion in the left high frontoparietal lobe likely to suggest recurrent brain abscess with diameters of 2.1 x 3.2 x 3.5 mm. Ceftriaxone (100 mg/kg/body weight every 12 hours), Vancomycin (20 mg/kg/body weight every six hours), and Metronidazole (10 mg/kg/body weight every eight hours) were started empirically. Left frontoparietal craniotomy and excision of the abscess were done. On examination, the abscess was thick-walled in the subcortical region extending deeper and posteriorly. At the posterior part, the abscess cavity was left deliberately to protect the motor cortex.

**Figure 3:**
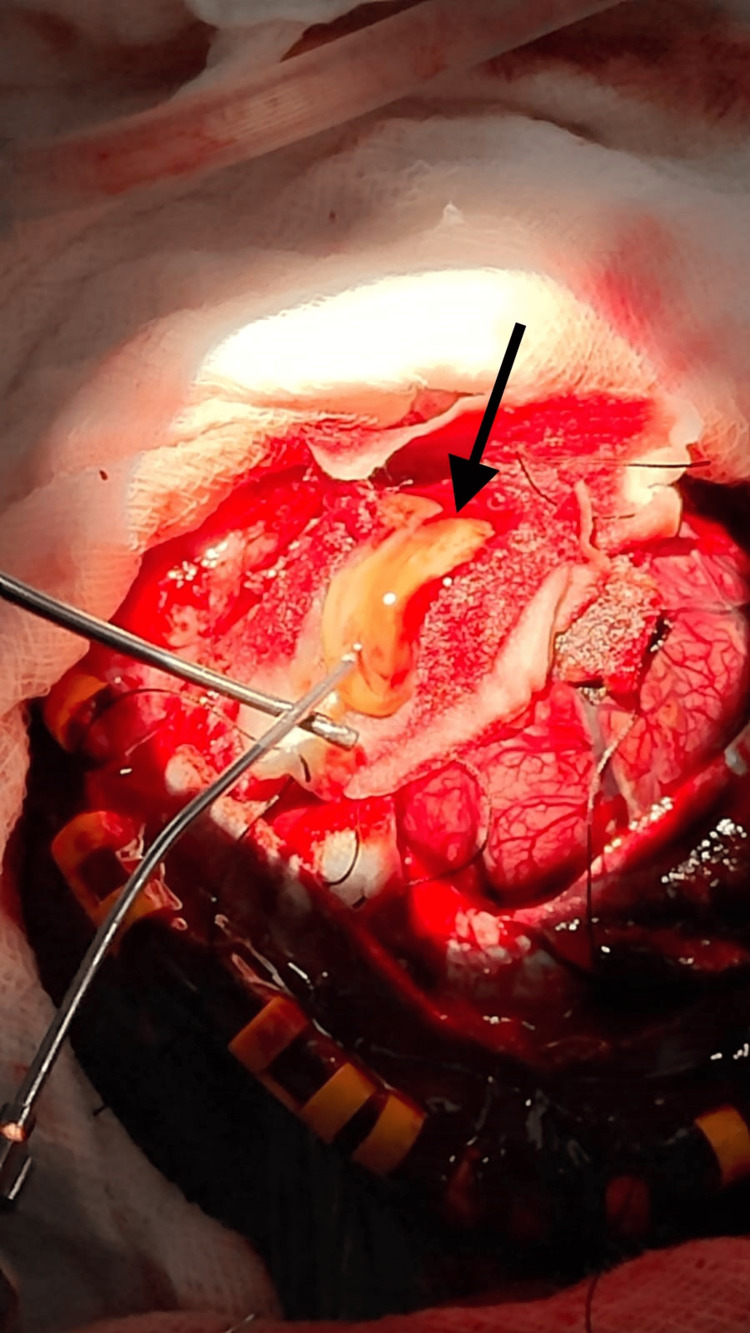
Large brain abscess being excised

The CSF culture taken on admission did not grow any organisms. The culture of the aspirated pus was found to grow AmpC beta-lactamase (class C of Ambler's classification of beta-lactamase) producing *Pseudomonas aeruginosa*. Antibiotics were revised according to the sensitivity of the culture to meropenem (40 mg/kg/body weight every eight hours). Intraoperatively the patient had supraventricular tachycardia (180 beats/min) which was treated with adenosine and lignocaine after which the rhythm was normal. The patient had persistent hypotension on the operative table hence an infusion of nor-epinephrine (0.1 mcg/kg/min) was started after giving two fluid boluses at 20 ml/kg. The infusion rate of nor-epinephrine was increased and vasopressin (0.2 mU/kg/min) infusion was started as the patient had persistent hypotension. Surgery was successful. The patient had no further hypotensive episodes and over a period of 24 hours, ionotropic supports were tapered and weaned off. Three days postoperatively the patient had one episode of focal convulsion involving the right-sided upper and lower extremities. Intravenous phenytoin was loaded (20 mg/kg) hence convulsions were controlled. And intravenous phenytoin was given at a maintenance dose of 5 mg/kg. A repeat CT scan of the head revealed subdural haemorrhage in the left frontoparietal lobe and postoperative oedema in the left frontoparietal lobe. Intravenous dexamethasone at 2 mg/kg was given to relieve the symptoms of cerebral oedema. The treatment with intravenous antibiotics was continued for four weeks and the patient showed significant clinical improvement as she had no further episodes of convulsions and her sensorium was well improved. Metronidazole was discontinued after four weeks, otherwise, meropenem and vancomycin continued for another two weeks. After three months of admission, the patient had a significant gain of power in all four limbs and hence was successfully discharged (Figure 3).

## Discussion

Brain abscesses have become a relatively rare entity and are mostly encountered in children between the age of four to 10 years. It is frequently encountered in children with uncorrected or partially corrected cyanotic congenital heart diseases. The right-to-left shunting present in cyanotic heart diseases, allows bacteria colonizing the airway to pass through the cerebral circulation [4]. These patients develop polycythaemia which further results in tissue hypoxia and ischaemia creating a suitable environment for the growth of bacteria. It is still a serious and potentially fatal entity leading to mortality and morbidity if not managed properly. Multiple and recurrent abscesses have a poorer prognosis [5]. However, with the emergence of newer neurosurgical techniques like stereotactic biopsy and aspiration and the availability of newer generations of antibiotics and modern neuroimaging techniques have contributed to better outcomes for brain abscesses.

The success of treatment lies in timely management with targeted antimicrobial therapy and early diagnosis of abscess and surgical drainage [6]. Factors associated with poor prognosis with high mortality rates include delayed administration of antibiotics at the time of admission, multiple and recurrent abscesses, younger age, meningitis, lesions large in size or near the ventricles, and if surgical aspiration is not implemented. In the case of a surgically treated abscess, antibiotics for four to six weeks are recommended and in the case of large multiple abscesses or an abscess treated solely medically, six to eight weeks of antimicrobial therapy is recommended [8]. Three to 12 months of antibiotic treatment is required for immunocompromised. A broad-spectrum antibiotic that can readily cross the blood-brain barrier or blood-CSF barrier should be used. Third-generation cephalosporins and metronidazole are commonly used. Metronidazole readily penetrates brain abscesses and has good bactericidal activity against anaerobes. Small abscesses less than 2.5 cm or multiple abscesses may be treated medically with follow-up scans to ensure a decrease in abscess size. Surgical excision of abscess is not routinely performed and is rarely needed as this is associated with greater morbidity compared to aspiration and is associated with the risk of rupture of abscess into the ventricular system [9]. These days aspiration of brain abscess has become the preferred method of drainage providing rapid relief from symptoms of raised intracranial pressure. It is considered easy to perform but has its own disadvantages being the requirement of repeated procedures. In 2009, Mut et al [10], compared the efficacy of aspiration versus the excision of the abscess and found more residual/recurrence cases in the aspiration group who needed a second aspiration, and no residual/recurrence was observed in the excision group [10]. Duration of hospital stay was comparatively less in the excision group than in the aspiration group.

In our patient, surgical excision of the abscess was successfully performed with the patient showing good results in the form of gain of power in the limbs and no further seizure episodes eventually over a period of four weeks. The posterior part of the cavity was left to protect the motor cortex. Excision of abscesses carries a risk of rupture of the abscess into the ventricular cavity. In a multivariate logistic analysis done in Japan, rupture of the abscess was a strong risk factor for poor outcomes [11]. In our patient, the abscess was located far from the ventricles and measured 3.5 cm in diameter. The abscess was successfully excised without any long-term neurological sequelae in the patient. Felsenstein et al. reported the factors for poorer outcomes in a child with a brain abscess. They include (1) age younger than five years, (2) Glasgow Coma Scale (GCS) score <8 at the time of admission [12]. The reason for the good outcome in our patient may be attributed to the timely and prompt initiation of antibiotics, good GCS at the time of admission, and the non-development of meningitis. 

## Conclusions

In conclusion, a seven-year-old girl, a diagnosed case of transposition of great arteries with a ventricular septal defect, developed a recurrent brain abscess. She was managed successfully, with good neurological outcomes, due to the timely initiation of antibiotics and excision.
